# Seasonal trends in antidepressant prescribing, depression, anxiety and self-harm in adolescents and young adults: an open cohort study using English primary care data

**DOI:** 10.1136/bmjment-2023-300855

**Published:** 2023-11-01

**Authors:** Ruth H Jack, Rebecca M Joseph, Chris Hollis, Julia Hippisley-Cox, Debbie Butler, Dave Waldram, Carol Coupland

**Affiliations:** 1 Centre for Academic Primary Care, Lifespan and Population Health, School of Medicine, University of Nottingham, Nottingham, UK; 2 National Institute for Health and Care Research Nottingham Biomedical Research Centre, Nottingham University Hospitals NHS Trust, Nottingham, UK; 3 National Institute for Health and Care Research MindTech MedTech Co-operative, Institute of Mental Health, School of Medicine, University of Nottingham, Nottingham, UK; 4 Nuffield Department of Primary Care Health Sciences, Radcliffe Observatory Quarter, University of Oxford, Oxford, UK

**Keywords:** child & adolescent psychiatry, depression & mood disorders, anxiety disorders, suicide & self-harm

## Abstract

**Background:**

There is an increasing demand for mental health services for young people, which may vary across the year.

**Objective:**

To determine whether there are seasonal patterns in primary care antidepressant prescribing and mental health issues in adolescents and young adults.

**Methods:**

This cohort study used anonymised electronic health records from general practices in England contributing to QResearch. It included 5 081 263 males and females aged 14–18 (adolescents), 19–23 and 24–28 years between 2006 and 2019. The incidence rates per 1000 person-years and the incidence rate ratios (IRRs) were calculated for the first records of a selective serotonin reuptake inhibitor (SSRI) prescription, depression, anxiety and self-harm. The IRRs were adjusted for year, region, deprivation, ethnic group and number of working days.

**Findings:**

There was an increase in SSRI prescribing, depression and anxiety incidence in male and female adolescents in the autumn months (September–November) that was not seen in older age groups. The IRRs for SSRI prescribing for adolescents peaked in November (females: 1.75, 95% CI 1.67 to 1.83, p<0.001; males: 1.72, 95% CI 1.61 to 1.84, p<0.001, vs in January) and for depression (females: 1.29, 95% CI 1.25 to 1.33, p<0.001; males: 1.29, 95% CI 1.23 to 1.35, p<0.001). Anxiety peaked in November for females aged 14–18 years (1.17, 95% CI 1.13 to 1.22, p<0.001) and in September for males (1.19, 95% CI 1.12 to 1.27, p<0.001).

**Conclusions:**

There were higher rates of antidepressant prescribing and consultations for depression and anxiety at the start of the school year among adolescents.

**Clinical implications:**

Support around mental health issues from general practitioners and others should be focused during autumn.

**WHAT IS ALREADY KNOWN ON THIS TOPIC**

- In recent years, there has been an increase in mental health issues and antidepressant prescribing in children and young people; however, specialist mental health services in the UK are currently struggling to meet this increased demand.
- There is little evidence on the patterns of these issues within a year, particularly among adolescents.

**WHAT THIS STUDY ADDS**

- Our large observational study of over five million adolescents and young adults shows that the rates of antidepressant prescribing, depression and anxiety have strong seasonal patterns among adolescents aged 14–18 years, but not among adults aged 19–23 years or 24–28 years, with the highest rates seen in autumn.
- Absolute rates were higher in females than in males within all age groups, but relative rates by month were similar in both sexes.

**HOW THIS STUDY MIGHT AFFECT RESEARCH, PRACTICE OR POLICY**

- General practitioners (GPs), teachers, social workers and others who support adolescents should concentrate resources around the autumn period, while future research should examine whether these patterns are also seen in younger children and those who do not present to GPs.

## Background

There has been concern that mental health issues in young people have been increasing in recent years. The incidence rates of depression, anxiety and self-harm have all been reported to be increasing in both males and females^1–4^ and that antidepressant prescriptions in adolescents have increased since 2005.^5–9^ In the last national survey of mental health in children and young people in England before the COVID-19 pandemic, 9% of those aged 11–16 years and 15% of those aged 17–19 years reported having any emotional disorder.^10^ Worldwide, around 10% of adolescents have reported self-harming,^11^ and rates of self-harm that leads to presentation at hospital emergency departments in England are highest in females aged 15–24 years.^12^

With this increase in mental health issues, services are increasingly coming under pressure to meet this growing need. The number of children referred to the National Health Service’s mental health services in England increased by 35% between 2018/2019 and 2019/2020, while the number of those having at least two contacts only increased by 4%.^13^ Understanding whether there are periods during the year when there will be an increased demand for mental health services can help with resource planning. There have been several studies in Canada examining the seasonal patterns of depression. One found that more depression occurred in the winter than in summer, with a small peak in September among those aged 12–24 years, which may be associated with the start of the academic year.^14^ Another study showed that eight depression symptoms had higher rates in the winter among those aged 12–24 years, while this pattern was only seen for sleep habits and appetite in adults aged 25 years and over.^15^ A systematic review found a possible impact of season on clinical depression, with higher rates found in the winter, but not on other measures of depression.^16^ In the Netherlands, winter has also been shown to be when adults are more likely to start antidepressant prescriptions from primary care.^17^ In the USA, self-poisoning suicide attempts are statistically significantly higher during the school months among those aged 10–18 years, but not among those aged 19–25 years.^18^ So far, there have been few studies examining the seasonal patterns of these issues in adolescents, and when adults have been studied, there is no consistent pattern . Previous work has also focused on surveys^14 15^ or hospital admissions,^16^ and there is a lack of population-based evidence on the seasonality of mental health conditions in adolescents.

### Objective

This study aimed to determine whether there are seasonal patterns in primary care antidepressant prescribing and consultations for mental health issues in adolescents and young adults by examining their first records of selective serotonin reuptake inhibitor (SSRI) prescriptions, anxiety, depression and self-harm from routinely collected primary care health records.

## Methods

### Data sources

Information was extracted from anonymised primary care electronic health records of a large primary care database, QResearch (V.45), linked to census data and Hospital Episode Statistics (HES) admitted patient care and outpatient data. At the time of the study, the QResearch database included the health records of over 38.6 million patients from general practices across the UK that record data using the Egton Medical Information Systems (EMIS) medical records computer system.

### Study participants

We defined the study’s open cohort as all males and females registered in the QResearch database in England who were aged 14–28 years between 1 January 2006 and 31 December 2019. The date of study entry was the latest date of the following: 12 months after their registration with a study practice, 12 months after the installation date of their practice’s EMIS computer system, 1 January of the year they turned 14 years old, or 1 January 2006. People were then followed up until the earliest date of them leaving the practice, 31 December the year before they turned 29 years old, death or the end of the follow-up period (31 December 2019).

### Outcomes

In order to look at new antidepressant prescribing and mental health issues, the outcomes for the analysis were the first prescription of an SSRI antidepressant and the first instances of consultations for depression, anxiety and self-harm recorded in the primary care data. Clinical code lists (Read and EMIS codes) were used to identify mental health issues. These have been reviewed by the research team (including a practising general practitioner, mental health specialists and epidemiologists with experience in UK electronic health records) and have been used in previous work.^19^ We only examined SSRIs as these are the most commonly prescribed antidepressants in adolescents^5^ and are recommended as first-line antidepressants for children and adults with depression.^20 21^ We also examined prescriptions for individual SSRIs (citalopram, sertraline or fluoxetine) separately. If the first record of each outcome was before the person’s study entry date, we excluded the participant from the analysis of that outcome. For the prescribing analysis, follow-up was censored on the first date of any antidepressant prescription (including non-SSRI antidepressants); for the other outcomes, follow-up was censored on the date when the outcome was first recorded.

### Covariates

We analysed six groups defined by age (14–18 years, 19–23 years and 24–28 years) and sex (male and female). We used the Office for National Statistics’ classification of the nine regions of England. We measured deprivation using the Townsend deprivation index, an area-based measure of deprivation that combines information on four indicators (unemployment, non-car ownership, non-home ownership, household overcrowding) from the census.^22^ Areas are then divided into quintiles based on their score. For ethnicity, we used the five broad ethnic groups from the England and Wales 2001 census: white (British, Irish, White other), Mixed (White and Black Caribbean, White and Black African, White and Asian, Mixed other), Asian or Asian British (Indian, Pakistani, Bangladeshi, any other Asian background), Black or Black British (Caribbean, African, any other Black background), and Chinese or other ethnic group, plus a not known category for those without an ethnicity recorded. When information on ethnicity was missing in the QResearch primary care records, we used the most recent, valid recorded ethnic group from the HES admitted patient care and outpatient data where available.

### Statistical analysis

We described the characteristics of the whole study population including the number and the proportion who had a first record of each of the outcomes within the study period. We combined the number of the first events and person-years in each month across the study period to calculate the incidence rates per 1000 person-years separately by age and sex group. To calculate the incidence rate ratios for each month in the six age–sex groups, we used Poisson regression with January as the baseline month. We took the number of working days in each month into account by multiplying the number of events by the number of working days divided by the total number of days in each month and used this adjusted count in the regression model. We additionally included terms for year, region, deprivation and ethnic group in the models. All analyses were carried out using Stata MP V.17.

### Patient and public involvement statement

The research question was suggested by the Nottingham GenerationR Young Person’s Advisory Group (YPAG). We subsequently presented this idea to seven young people from the NeurOx YPAG, a group based in Oxford with an interest in mental health research. The results were then discussed with five young people in a second session with the NeurOx YPAG. We also presented and distributed the results to the Nottingham GenerationR YPAG. Updates on the project were also discussed in our regular project meetings, which included two public contributors who are coauthors of the paper.

## Findings

The study cohort included 5 081 263 males and females aged 14–28 years, with a total follow-up time of over 17.9 million person-years. Table 1 shows the characteristics of the entire study population and the number of people with their outcomes first recorded during the study period while aged 14–28 years. Just over half (52.5%) of the study population were female, 28.4% lived in London and a quarter (25.1%) lived in areas in the most deprived quintile of England. Ethnicity information was not known for 11.2% of the study population, and of those with a known ethnic group 76.0% were white.

**Table 1:** Characteristics of the entire study population aged 14–28 years, England, 2006–2019

	n (%)
Sex	
Female	2 665 681 (52.46)
Male	2 415 582 (47.54)
Region	
East Midlands	263 503 (5.19)
East of England	145 945 (2.87)
London	1 440 473 (28.35)
North East	166 704 (3.28)
North West	785 490 (15.46)
South East	1 057 045 (20.80)
South West	530 486 (10.44)
West Midlands	413 949 (8.15)
Yorkshire and Humber	277 668 (5.46)
Townsend deprivation quintile	
1 (least deprived)	788 343 (15.51)
2	887 389 (17.46)
3	972 339 (19.14)
4	1 140 155 (22.44)
5 (most deprived)	1 276 398 (25.12)
Not known	16 639 (0.33)
Ethnic group	
White	3 411 054 (67.13)
Mixed	86 356 (1.70)
Asian or Asian British	495 412 (9.75)
Black or black British	242 655 (4.78)
Chinese or other ethnic group	277 467 (5.46)
Not known	568 319 (11.18)
Known ethnic group	
White	3 411 054 (75.58)
Mixed	86 356 (1.91)
Asian or Asian British	495 412 (10.98)
Black or black British	242 655 (5.38)
Chinese or other ethnic group	277 467 (6.15)
Outcome first recorded at age 14–28 years	
SSRI	279 271 (5.50)
Citalopram	114 228 (2.25)
Fluoxetine	68 569 (1.35)
Sertraline	88 015 (1.73)
Depression	338 634 (6.66)
Anxiety	186 150 (3.66)
Self-harm	53 506 (1.05)

SSRI, selective serotonin reuptake inhibitor.

The overall incidence rates per 1000 person-years for all outcomes in the whole study period are shown in table 2. Overall, females aged 19–23 years had the highest incidence rates for all outcomes, apart from self-harm where the highest rates were in females aged 14–18 years. Within each age group, females had higher incidence rates for all outcomes than males. Fluoxetine was the most commonly first prescribed antidepressant in those aged 14–18 years, while in the older age groups the most commonly prescribed was citalopram.

**Table 2:** Overall incidence rates per 1000 person-years for each outcome by age and sex, England, 2006–2019

	14–18 years			19–23 years			24–28 years		
	Events	Person-years	Rate	Events	Person-years	Rate	Events	Person-years	Rate
Females									
SSRI	30 470	2 391 683	12.7	82 911	2 373 588	34.9	62 954	2 562 916	24.6
Citalopram	7374	2 391 683	3.1	36 505	2 373 588	15.4	28 801	2 562 916	11.2
Fluoxetine	14 626	2 391 683	6.1	17 788	2 373 588	7.5	12 285	2 562 916	4.8
Sertraline	8036	2 391 683	3.4	26 169	2 373 588	11.0	19 663	2 562 916	7.7
Depression	55 153	2 330 678	23.7	88 649	2 354 850	37.6	67 922	2 579 496	26.3
Anxiety	32 324	2 370 754	13.6	47 544	2 607 971	18.2	43 705	3 017 587	14.5
Self-harm	18 613	2 395 820	7.8	10 732	2 696 963	4.0	5777	3 180 782	1.8
Males									
SSRI	14 050	2 747 359	5.1	48 437	2 852 283	17.0	40 449	3 000 183	13.5
Citalopram	2960	2 747 359	1.1	20 230	2 852 283	7.1	18 358	3 000 183	6.1
Fluoxetine	6666	2 747 359	2.4	9931	2 852 283	3.5	7273	3 000 183	2.4
Sertraline	4156	2 747 359	1.5	16 686	2 852 283	5.9	13 305	3 000 183	4.4
Depression	26 245	2 718 992	9.7	55 435	2 841 080	19.5	45 230	3 030 836	14.9
Anxiety	14 083	2 737 576	5.1	25 431	2 971 614	8.6	23 063	3 267 201	7.1
Self-harm	5620	2 766 339	2.0	7293	3 034 125	2.4	5471	3 367 222	1.6

SSRI, selective serotonin reuptake inhibitor.

**Figure 1:**
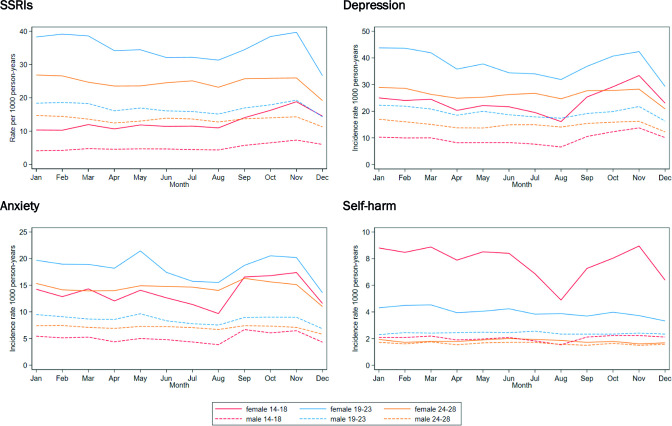
Incidence rates for SSRIs, depression, anxiety and self-harm per 1000 person-years for each month, by age and sex, England, 2006–2019. SSRIs, selective serotonin reuptake inhibitors.

Figure 1 shows the incidence rates per 1000 person-years for SSRI prescriptions, depression, anxiety and self-harm for each month by age and sex group. For males and females aged 14–18 years, SSRI prescribing increased in March, was fairly stable until August, then started to increase in September, peaking in November. The highest rates of SSRI prescribing were seen in January to March and in October and November in the 19–23 years age group. For those aged 24–28 years, the monthly SSRI prescribing rates were reasonably stable throughout the year. Patterns of individual antidepressants were similar to those seen for SSRIs overall (online supplemental figure S1), with the exception of females aged 14–18 years, who had the highest incidence rates of fluoxetine prescribing in November and December.

10.1136/bmjment-2023-300855.supp1 Supplementary data

The incidence rates of depression in males and females aged 14–18 years decreased between January and August, then increased, peaking in November. The incidence rates of depression were highest in January for males and females aged 19–23 years and 24–28 years, declined until August, then increased, with a final dip in December. The incidence rates of anxiety were highest in September to November in those aged 14–18 years and peaked in May in the 19–23 years age group. The incidence rates of self-harm were similar throughout the year for all age and sex groups, except for a large decrease in July and August for females aged 14–18 years.

**Figure 2:**
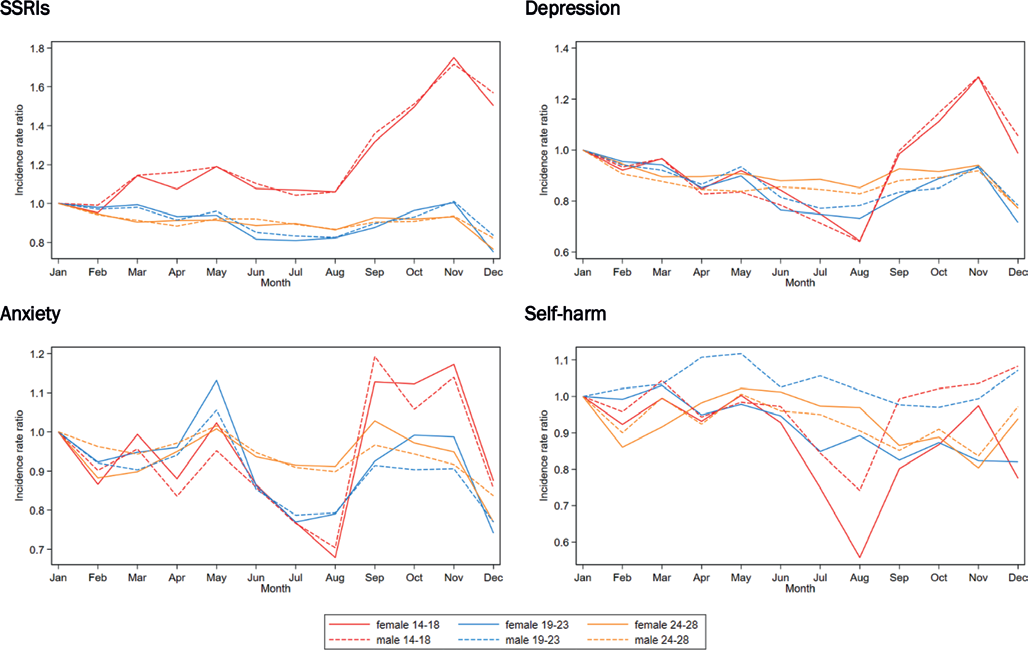
Incidence rate ratios for SSRIs, depression, anxiety and self-harm adjusted for year, region, deprivation, ethnic group and working days, for each month, by age and sex, England, 2006–2019. SSRIs, selective serotonin reuptake inhibitors.

Adjusted incidence rate ratios for SSRI prescribing, depression and anxiety in both males and females aged 14–18 years revealed an increase in the autumn months (September–November) compared with January (figure 2 and online supplemental tables 1 and 2). For females in this age group, the highest incidence rate ratios compared with January were in November for SSRI prescribing (1.75, 95% CI 1.67 to 1.83, p<0.001), depression (1.29, 95% CI 1.25 to 1.33, p<0.001) and anxiety (1.17, 95% CI 1.13 to 1.22, p<0.001). For males aged 14–18 years, the highest incidence rate ratios were also in November for SSRI prescribing (1.72, 95% CI 1.61 to 1.84, p<0.001) and depression (1.29, 95% CI 1.23 to 1.35, p<0.001), and in September for anxiety (1.19, 95% CI 1.12 to 1.27, p<0.001). In this age group, the incidence rate ratios for depression (0.64, 95% CI 0.61 to 0.68, p<0.001, in males; 0.64, 95% CI 0.62 to 0.67, p<0.001, in females), anxiety (0.70, 95% CI 0.66 to 0.76, p<0.001, in males; 0.68, 95% CI 0.65 to 0.71, p<0.001, in females) and self-harm (0.74, 95% CI 0.66 to 0.83, p<0.001, in males; 0.56, 95% CI 0.52 to 0.59, p<0.001, in females) were all lowest in August. Generally, very similar incidence rate ratio patterns for all outcomes were seen in the 19–23 years and 24–28 years age groups across the months. However, males aged 19–23 years (1.06, 95% CI 1.01 to 1.11, p=0.023) and females aged 19–23 years (1.13, 95% CI 1.09 to 1.17, p<0.001) both had an increase in the rate of anxiety in May, and there was an increase in the rate of self-harm in males aged 19–23 years in April (1.11, 95% CI 1.01 to 1.22, p=0.033) and May (1.12, 95% CI 1.02 to 1.23, p=0.019).

## Discussion

This study has shown that there is an increase in the incidence of SSRI prescribing, depression and anxiety in males and females aged 14–18 years in the autumn months and a peak in anxiety in May in those aged 19–23 years. In the 19–23 and 24–28 years age groups, the rates of SSRI prescribing and depression were highest in January, decreased in summer and only increased to earlier levels in autumn. This peak in consultations for mental health issues in adolescents occurs at the beginning of the academic school year, which in England starts in September, before the first major break in December. Self-harm rates varied less by month in all groups, apart from a decrease in the summer months in females aged 14–18 years.

### Strengths and limitations of the study

This study included information on more than five million adolescents and young adults from across England, with almost 18 million years of follow-up, making it the largest study so far to examine the seasonal trends in mental health and antidepressant prescribing. QResearch is a nationally representative database,^23^ suggesting the results would be generalisable across England. Using prospectively collected routine health data from the general population means the study is free from selection and recall bias. By including symptoms as well as diagnoses of mental health issues in our outcome definitions, there should be more timely and comprehensive recording of when people first visited their general practitioner (GP) for these issues, rather than waiting for more severe problems to persist. GPs have been increasingly using depression^24^ and anxiety^25^ symptoms rather than diagnosis codes.

A limitation of this study is that the information on prescribing and diagnosis came from primary care only. People who did not seek any help from health professionals will be missed from these analyses, suggesting that we may only have included more severe cases. It is unlikely that this would be affected by month, although people may find it more difficult to get appointments in the winter months when GPs tend to be busier.^26^ In this case, we may be underestimating the incidence during this period. GPs should be informed about secondary care diagnoses, and these would be included in GP records; however, antidepressant prescriptions made in secondary care would not have been captured in the primary care data used, and if care was transferred to primary care then subsequent prescriptions may have incorrectly been classed as the patient’s first. We did not distinguish between seasonal affective disorder (SAD) and other forms of depression. Some of the cases of depression may have been SAD, which would have affected the seasonal pattern found. Primary care is the most commonly contacted health professional service by children and young people with a mental health disorder for support in England.^10^ Teachers and schools can directly refer students to services such as child and adolescent mental health services (CAMHS), bypassing primary care. However, the number of children being referred to CAMHS has been increasing before the COVID-19 pandemic,^13^ meaning primary care would remain a more easily accessible healthcare option.

### Comparison with other studies

While a previous study based on national surveys in Canada found a peak in major depressive episodes for those aged 12–24 years in September,^14^ we found the first GP visits for depression continued to rise throughout autumn in adolescents, peaking in November. Generally, the increase in mental health issues previously found during the winter^14–16^ and school terms^18^ in other studies was replicated here, with our study able to take other important factors into account, such as deprivation and ethnicity.

In determining whether patterns in young people’s mental health might be associated with timing of events in the academic year, we were not able to distinguish those in the 19–23 years age group who were still in education. However, student status has not previously been associated with differences in depression^15^ or self-harm.^27^ The peak in anxiety rates we found in May coincides with a period of examinations in many higher education institutions. Our study found very similar results for the two young adult groups aged 19–23 years and 24–28 years. These differed from the patterns in the adolescents, suggesting that seasonal patterns vary between adolescents and young adults. Factors related to weather and daylight hours might be particularly affecting mental health in adolescents and young people, as well as factors related to education. However, this study is based on an observational study design and so we were unable to infer causal relationships.

In our discussions, the YPAG members suggested that adolescents may avoid seeking help when it is most needed due to the stresses of this period. For example, they might not believe that visiting a GP during exams (usually around May and June in England) is the best use of their time and therefore put it off until later in the year. GPs may regard exam periods as producing levels of depression and anxiety that are to be expected and be more likely to record such episodes when seen at different times of the year. GPs may also defer prescribing antidepressants until the mental health issues have persisted for a time. Schools and colleges may have more support and resources available to students during exam periods, leading to fewer people feeling the need to seek further help at these times. Finalising applications for university courses usually takes place during the autumn, and the pressure of this, along with mock exams, may be having an effect on mental health in adolescents.

Teachers play an important role in students’ mental health, with 49% of young people going to teachers for help in a survey from England.^28^ Teachers may advise adolescents with mental health issues to visit their GP, which would result in an increased demand at the start of the new school year and which may partially explain the dip seen during summer. Academic settings are convenient for adolescents to seek help for issues that are concerning them, although the YPAG we spoke to highlighted that speaking to trained specialists is preferable.

This study was not designed to determine the reasons why different seasonal patterns are seen throughout the year in different age groups, and there could be several explanations, including combinations of factors for the patterns we have found. Qualitative studies where adolescents can discuss their experiences of mental health would provide direct evidence about which elements have affected their mental health and help-seeking, along with a better understanding of how they can be best supported when needed. Studies gaining insights from teachers and other education staff may also point towards ways schools and colleges can help students when it is most needed. Further research comparing the results from our study with other countries, including those in the southern hemisphere, could help separate the effects of academic events and seasons. Intervention studies to see whether concentrating school mental health sessions in the first term, when the incidence rates of SSRI prescribing, depression and anxiety in males and females aged 14–18 years are highest, or other activities are beneficial may also be useful.

## Clinical implications

GPs and other healthcare providers should focus their resources during autumn when there is a higher demand for mental health services from adolescents.

## Data Availability

Data may be obtained from a third party and are not publicly available. Access to QResearch data is according to the information on the QResearch website (www.qresearch.org).
