# Supplementary material for: Seasonal trends in antidepressant prescribing, depression, anxiety and self-harm in adolescents and young adults: an open cohort study using English primary care data
Source: BMJ Ment Health. 2023 Nov 1;26(1):e300855. doi: 10.1136/bmjment-2023-300855 (PMC10649373; doi:10.1136/bmjment-2023-300855)
Supplement: Supplementary data [file bmjment-2023-300855supp001.pdf]

Supplementary Fig 1 Incidence rates for citalopram, sertraline, and fluoxetine per 1000 person-years and incidence rate ratios for each month, by age and sex, England, 2006-2019. Incidence rate ratios for each month compared to January and adjusted for year, region, deprivation, ethnic group and working days

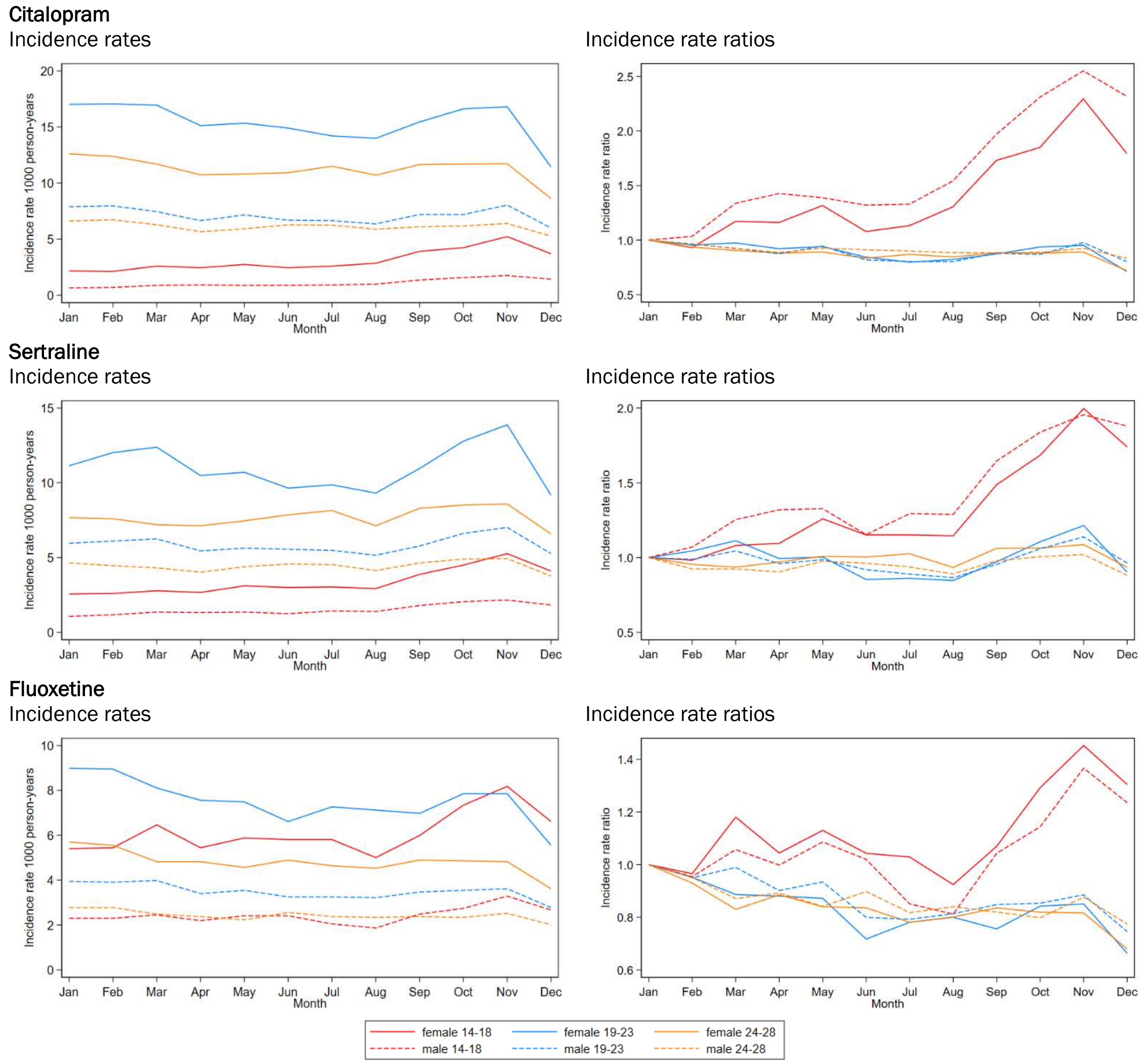

Supplementary Table 1: Female incidence rate ratios, 95% confidence intervals (CI) and p values for SSRIs, depression, anxiety and self-harm adjusted for year, region, deprivation, ethnic group and working days, for each month, by age group, England, 2006-2019

	14-18 years			19-23 years			24-28 years		
	IRR	(95% CI)	p	IRR	(95% CI)	p	IRR	(95% CI)	p
SSRI prescriptions									
Jan	1.00			1.00			1.00		
Feb	0.95	(0.90-1.00)	0.065	0.98	(0.95-1.01)	0.124	0.95	(0.92-0.98)	<0.001
Mar	1.14	(1.09-1.20)	<0.001	0.99	(0.97-1.02)	0.603	0.90	(0.88-0.93)	<0.001
Apr	1.07	(1.02-1.13)	0.005	0.93	(0.91-0.96)	<0.001	0.91	(0.88-0.94)	<0.001
May	1.19	(1.14-1.25)	<0.001	0.94	(0.91-0.96)	<0.001	0.91	(0.89-0.94)	<0.001
Jun	1.08	(1.02-1.13)	0.004	0.82	(0.79-0.84)	<0.001	0.89	(0.86-0.91)	<0.001
Jul	1.07	(1.02-1.12)	0.007	0.81	(0.79-0.83)	<0.001	0.90	(0.87-0.92)	<0.001
Aug	1.06	(1.01-1.11)	0.021	0.82	(0.80-0.85)	<0.001	0.86	(0.84-0.89)	<0.001
Sep	1.32	(1.26-1.38)	<0.001	0.88	(0.85-0.90)	<0.001	0.93	(0.90-0.96)	<0.001
Oct	1.50	(1.43-1.57)	<0.001	0.96	(0.94-0.99)	0.006	0.92	(0.89-0.95)	<0.001
Nov	1.75	(1.67-1.83)	<0.001	1.00	(0.98-1.03)	0.755	0.93	(0.90-0.96)	<0.001
Dec	1.50	(1.44-1.57)	<0.001	0.75	(0.73-0.77)	<0.001	0.76	(0.74-0.79)	<0.001
Depression									
Jan	1.00			1.00			1.00		
Feb	0.92	(0.89-0.95)	<0.001	0.95	(0.93-0.98)	<0.001	0.95	(0.92-0.97)	<0.001
Mar	0.97	(0.93-1.00)	0.038	0.94	(0.92-0.97)	<0.001	0.90	(0.87-0.92)	<0.001
Apr	0.85	(0.82-0.88)	<0.001	0.85	(0.83-0.87)	<0.001	0.90	(0.87-0.92)	<0.001
May	0.92	(0.89-0.95)	<0.001	0.90	(0.88-0.92)	<0.001	0.91	(0.88-0.93)	<0.001
Jun	0.84	(0.81-0.87)	<0.001	0.76	(0.74-0.79)	<0.001	0.88	(0.85-0.91)	<0.001
Jul	0.75	(0.72-0.78)	<0.001	0.75	(0.73-0.77)	<0.001	0.89	(0.86-0.91)	<0.001
Aug	0.64	(0.62-0.67)	<0.001	0.73	(0.71-0.75)	<0.001	0.85	(0.83-0.88)	<0.001
Sep	0.98	(0.95-1.02)	0.365	0.82	(0.80-0.84)	<0.001	0.93	(0.90-0.95)	<0.001
Oct	1.11	(1.08-1.15)	<0.001	0.89	(0.87-0.91)	<0.001	0.92	(0.89-0.94)	<0.001
Nov	1.29	(1.25-1.33)	<0.001	0.93	(0.91-0.96)	<0.001	0.94	(0.91-0.97)	<0.001
Dec	0.99	(0.96-1.02)	0.431	0.72	(0.70-0.74)	<0.001	0.77	(0.75-0.79)	<0.001
Anxiety									
Jan	1.00			1.00			1.00		
Feb	0.87	(0.83-0.91)	<0.001	0.92	(0.89-0.96)	<0.001	0.88	(0.85-0.92)	<0.001
Mar	0.99	(0.95-1.04)	0.780	0.95	(0.91-0.98)	0.003	0.90	(0.87-0.93)	<0.001
Apr	0.88	(0.84-0.92)	<0.001	0.96	(0.93-1.00)	0.028	0.95	(0.92-0.99)	0.007
May	1.02	(0.98-1.07)	0.290	1.13	(1.09-1.17)	<0.001	1.01	(0.97-1.05)	0.674
Jun	0.87	(0.83-0.90)	<0.001	0.86	(0.83-0.90)	<0.001	0.94	(0.90-0.97)	<0.001
Jul	0.77	(0.73-0.80)	<0.001	0.77	(0.74-0.80)	<0.001	0.92	(0.88-0.95)	<0.001
Aug	0.68	(0.65-0.71)	<0.001	0.79	(0.76-0.82)	<0.001	0.91	(0.88-0.95)	<0.001
Sep	1.13	(1.08-1.18)	<0.001	0.93	(0.89-0.96)	<0.001	1.03	(0.99-1.07)	0.134
Oct	1.12	(1.08-1.17)	<0.001	0.99	(0.96-1.03)	0.637	0.97	(0.94-1.01)	0.132
Nov	1.17	(1.13-1.22)	<0.001	0.99	(0.95-1.02)	0.474	0.95	(0.91-0.99)	0.006
Dec	0.88	(0.84-0.91)	<0.001	0.74	(0.71-0.77)	<0.001	0.77	(0.74-0.80)	<0.001
Self-harm									
Jan	1.00			1.00			1.00		
Feb	0.92	(0.87-0.98)	0.006	0.99	(0.92-1.07)	0.834	0.86	(0.77-0.96)	0.006
Mar	0.99	(0.94-1.05)	0.848	1.03	(0.96-1.11)	0.433	0.92	(0.83-1.02)	0.096
Apr	0.93	(0.88-0.99)	0.013	0.95	(0.88-1.02)	0.176	0.98	(0.89-1.09)	0.733
May	1.00	(0.95-1.06)	0.912	0.98	(0.91-1.05)	0.560	1.02	(0.92-1.13)	0.667
Jun	0.93	(0.88-0.98)	0.009	0.95	(0.88-1.02)	0.155	1.01	(0.91-1.12)	0.814
Jul	0.75	(0.71-0.79)	<0.001	0.85	(0.79-0.92)	<0.001	0.97	(0.88-1.08)	0.599
Aug	0.56	(0.52-0.59)	<0.001	0.89	(0.83-0.96)	0.003	0.97	(0.88-1.07)	0.547
Sep	0.80	(0.76-0.85)	<0.001	0.83	(0.76-0.89)	<0.001	0.87	(0.78-0.96)	0.007
Oct	0.87	(0.82-0.92)	<0.001	0.87	(0.81-0.94)	<0.001	0.89	(0.80-0.99)	0.025
Nov	0.97	(0.92-1.03)	0.352	0.82	(0.76-0.89)	<0.001	0.80	(0.72-0.89)	<0.001
Dec	0.78	(0.73-0.82)	<0.001	0.82	(0.76-0.88)	<0.001	0.94	(0.85-1.04)	0.226

Supplementary Table 2: Male incidence rate ratios, 95% confidence intervals (CI) and p values for SSRIs, depression, anxiety and self-harm adjusted for year, region, deprivation, ethnic group and working days, for each month, by age group, England, 2006-2019

	14-18 years			19-23 years			24-28 years		
	IRR	(95% CI)	p	IRR	(95% CI)	p	IRR	(95% CI)	p
SSRI prescriptions									
Jan	1.00			1.00			1.00		
Feb	0.99	(0.92-1.07)	0.805	0.97	(0.94-1.01)	0.097	0.94	(0.90-0.98)	0.002
Mar	1.14	(1.06-1.23)	<0.001	0.98	(0.95-1.01)	0.251	0.91	(0.88-0.95)	<0.001
Apr	1.16	(1.08-1.25)	<0.001	0.91	(0.88-0.95)	<0.001	0.88	(0.85-0.92)	<0.001
May	1.19	(1.11-1.28)	<0.001	0.96	(0.93-0.99)	0.022	0.92	(0.89-0.96)	<0.001
Jun	1.10	(1.02-1.19)	0.010	0.85	(0.82-0.88)	<0.001	0.92	(0.89-0.96)	<0.001
Jul	1.04	(0.97-1.12)	0.274	0.83	(0.80-0.86)	<0.001	0.89	(0.86-0.93)	<0.001
Aug	1.06	(0.98-1.14)	0.122	0.82	(0.79-0.85)	<0.001	0.87	(0.83-0.90)	<0.001
Sep	1.36	(1.27-1.46)	<0.001	0.90	(0.87-0.93)	<0.001	0.90	(0.87-0.94)	<0.001
Oct	1.51	(1.41-1.62)	<0.001	0.93	(0.90-0.96)	<0.001	0.91	(0.87-0.94)	<0.001
Nov	1.72	(1.61-1.84)	<0.001	1.01	(0.98-1.05)	0.552	0.94	(0.90-0.97)	<0.001
Dec	1.57	(1.47-1.68)	<0.001	0.83	(0.80-0.86)	<0.001	0.82	(0.79-0.85)	<0.001
Depression									
Jan	1.00			1.00			1.00		
Feb	0.93	(0.89-0.98)	0.007	0.94	(0.91-0.97)	<0.001	0.91	(0.87-0.94)	<0.001
Mar	0.97	(0.92-1.01)	0.152	0.92	(0.89-0.95)	<0.001	0.88	(0.85-0.91)	<0.001
Apr	0.83	(0.79-0.87)	<0.001	0.87	(0.84-0.89)	<0.001	0.85	(0.82-0.88)	<0.001
May	0.84	(0.80-0.88)	<0.001	0.94	(0.91-0.97)	<0.001	0.84	(0.81-0.87)	<0.001
Jun	0.78	(0.74-0.82)	<0.001	0.81	(0.79-0.84)	<0.001	0.86	(0.82-0.89)	<0.001
Jul	0.71	(0.68-0.75)	<0.001	0.77	(0.75-0.80)	<0.001	0.85	(0.82-0.88)	<0.001
Aug	0.64	(0.61-0.68)	<0.001	0.78	(0.76-0.81)	<0.001	0.83	(0.80-0.86)	<0.001
Sep	1.00	(0.95-1.05)	0.951	0.84	(0.81-0.86)	<0.001	0.88	(0.85-0.91)	<0.001
Oct	1.15	(1.09-1.20)	<0.001	0.85	(0.82-0.88)	<0.001	0.89	(0.86-0.93)	<0.001
Nov	1.29	(1.23-1.35)	<0.001	0.94	(0.91-0.97)	<0.001	0.92	(0.89-0.95)	<0.001
Dec	1.06	(1.01-1.11)	0.024	0.78	(0.76-0.81)	<0.001	0.77	(0.74-0.80)	<0.001
Anxiety									
Jan	1.00			1.00			1.00		
Feb	0.90	(0.84-0.97)	0.003	0.92	(0.88-0.97)	0.001	0.96	(0.91-1.01)	0.159
Mar	0.96	(0.90-1.02)	0.185	0.90	(0.86-0.95)	<0.001	0.94	(0.90-0.99)	0.026
Apr	0.84	(0.78-0.90)	<0.001	0.94	(0.90-0.99)	0.013	0.97	(0.92-1.02)	0.277
May	0.95	(0.89-1.02)	0.144	1.06	(1.01-1.11)	0.023	1.02	(0.97-1.07)	0.546
Jun	0.86	(0.80-0.92)	<0.001	0.85	(0.81-0.90)	<0.001	0.95	(0.90-1.00)	0.041
Jul	0.77	(0.71-0.82)	<0.001	0.79	(0.75-0.83)	<0.001	0.91	(0.86-0.96)	<0.001
Aug	0.70	(0.66-0.76)	<0.001	0.79	(0.75-0.83)	<0.001	0.90	(0.85-0.95)	<0.001
Sep	1.19	(1.12-1.27)	<0.001	0.91	(0.87-0.96)	<0.001	0.97	(0.92-1.02)	0.198
Oct	1.06	(0.99-1.13)	0.085	0.90	(0.86-0.95)	<0.001	0.94	(0.90-0.99)	0.026
Nov	1.14	(1.07-1.21)	<0.001	0.91	(0.86-0.95)	<0.001	0.92	(0.87-0.97)	0.001
Dec	0.86	(0.80-0.92)	<0.001	0.77	(0.73-0.81)	<0.001	0.84	(0.79-0.88)	<0.001
Self-harm									
Jan	1.00			1.00			1.00		
Feb	0.96	(0.86-1.07)	0.436	1.02	(0.93-1.13)	0.663	0.90	(0.81-1.00)	0.059
Mar	1.04	(0.94-1.16)	0.415	1.03	(0.94-1.14)	0.491	1.00	(0.90-1.10)	0.939
Apr	0.94	(0.85-1.05)	0.286	1.11	(1.01-1.22)	0.033	0.92	(0.83-1.03)	0.147
May	0.98	(0.89-1.09)	0.765	1.12	(1.02-1.23)	0.019	1.01	(0.91-1.12)	0.903
Jun	0.97	(0.87-1.08)	0.600	1.03	(0.93-1.13)	0.606	0.96	(0.86-1.07)	0.446
Jul	0.84	(0.76-0.94)	0.002	1.06	(0.96-1.16)	0.247	0.95	(0.86-1.06)	0.341
Aug	0.74	(0.66-0.83)	<0.001	1.02	(0.92-1.12)	0.739	0.91	(0.81-1.01)	0.067
Sep	0.99	(0.89-1.10)	0.901	0.98	(0.89-1.08)	0.630	0.85	(0.76-0.95)	0.004
Oct	1.02	(0.92-1.13)	0.684	0.97	(0.88-1.07)	0.527	0.91	(0.82-1.01)	0.080
Nov	1.04	(0.93-1.15)	0.510	0.99	(0.90-1.09)	0.893	0.84	(0.75-0.93)	0.001
Dec	1.08	(0.98-1.20)	0.121	1.07	(0.98-1.18)	0.137	0.97	(0.88-1.08)	0.587
